# Regional assessment of left ventricular torsion by CMR tagging

**DOI:** 10.1186/1532-429X-10-26

**Published:** 2008-05-27

**Authors:** Iris K Rüssel, Marco J Götte, Joost P Kuijer, J Tim Marcus

**Affiliations:** 1 Dept. of Physics and Medical Technology, Vrije Universiteit Medical Center, De Boelelaan 1118 1081 HV Amsterdam, the Netherlands; 2 Dept. of Cardiology, Vrije Universiteit Medical Center, Amsterdam, the Netherlands

## Abstract

**Purpose:**

To introduce a standardized method for calculation of left ventricular torsion by CMR tagging and to determine the accuracy of torsion analysis in regions using an analytical model.

**Methods:**

Torsion between base and apex, base and mid, and mid and apex levels was calculated using CSPAMM tagging and Harmonic Phase tracking. The accuracy of torsion analysis on a regional basis (circumferential segments and transmural layers) was analyzed using an analytical model of a deforming cylinder with a displaced axis of rotation (AoR). Regional peak torsion values from twelve healthy volunteers calculated by the described method were compared to literature.

**Results:**

The deviation from the analytical torsion per % AoR-displacement (of the radius) was 0.90 ± 0.44% for the circumferential segments and only 0.05% for the transmural layers. In the subjects, circumferentially, anterolateral torsion was larger than inferior (12.4 ± 3.9° vs. 5.0 ± 3.3°, N.S.). Transmurally, endocardial torsion was smaller than epicardial (7.5 ± 1.3° vs. 8.0 ± 1.5°, p < 0.001).

**Conclusion:**

Variability in the position of the AoR causes a large variability in torsion in circumferential segments. This effect was negligible for global torsion, and torsion calculated in transmural layers. Results were documented for the healthy human heart and are in agreement with data from literature.

## Introduction

Torsion is the wringing motion induced by contracting myofibers in the left ventricular (LV) wall, in order to eject blood from the ventricle. The subsequent fast untwisting during early diastole is a major indicator of the restoring forces that contribute to rapid filling, since the blood is actively sucked from the left atrium [1] into the LV.

Subendo- and subepicardial myofibers are obliquely oriented in opposite directions, with a smooth, transmural transition between the fiber directions [2]. Since torsion is directly related to myofiber orientation, structure, and function, it is an important indicator for the condition of the heart. Torsion was found to be a sensitive marker for both systolic and diastolic dysfunction [3-10]. This may allow for an early differentiation between diseased and normal myocardium, and discrimination between systolic and diastolic heart failure.

**Figure 1:**
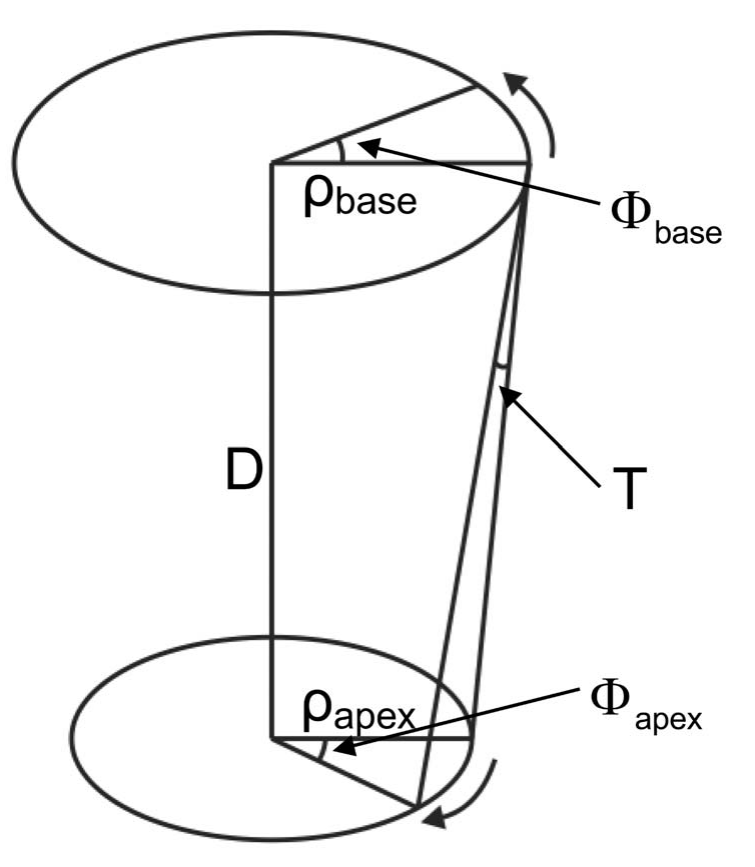
A sketch of a basal and an apical plane and the torsional deformation. Twist is defined as (Φ_*apex *_- Φ_*base*_), the twist per unit length as (Φ_*apex *_- Φ_*base*_). ρ_*m *_/*D *and the torsion T (CL shear angle) as (Φ_*apex *_- Φ_*base*_). ρ_m_/*D*, with ρ_m _the mean radius of the basal and apical level, in the undeformed and the deformed state. Counterclockwise rotation as seen from the apex is positive.

However, no uniform method to calculate torsion is yet available. There is still a debate on how to describe and analyze LV torsion in an optimal way. Several methods to describe torsion have been published [11-13]. Calculation of torsion as the circumferential-longitudinal (CL) shear angle, takes both the length and the radius of the heart into account (Fig. 1). Therefore, it allows for comparison between hearts of different sizes and is directly related to fiber orientation and the processes in the cardiac wall [14]. In this article, this definition is used as the torsion.

Furthermore, the question whether analysis of torsion on a regional basis is clinically useful, remains unanswered. In studies on healthy subjects, information on reference values of regional torsion is available [11,15,16], whereas in patient studies, data on regional torsion is scarce. The canine study by Buchalter et al. [17], showing that inferior wall ischemia influenced both inferior and anterior wall rotation, illustrates that torsion is a global measure of heart function.

**Figure 2:**
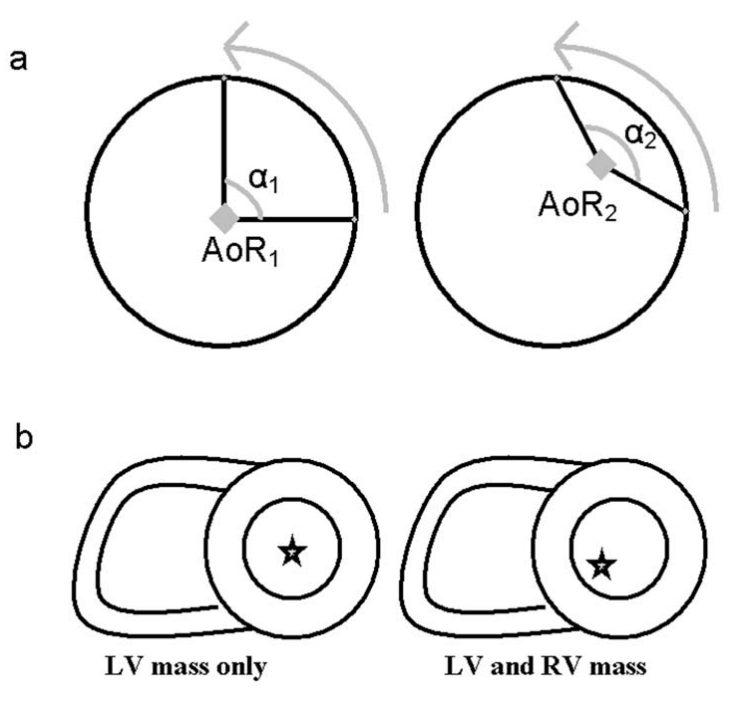
A displaced axis of rotation results in a different observed rotation angle for the same displacement (a). Inclusion of the RV mass in the calculation of the AoR as the center of mass will move the AoR more towards inferoseptal (b).

An important issue that may affect analysis of regional torsion is the definition of the axis of rotation (AoR). Regional differences in torsion might be explained by the placement of the AoR. Although not previously applied, it was suggested that the AoR might be the center of mass of both the LV and the right ventricle (RV) together [15,16], which places the point closer to the septum than when only the LV mass is taken into account (Fig. 2). Besides the uncertainty in definition, the accuracy of the AoR will also depend on correct contouring of the cardiac wall.

In this paper, a method is described that is comparable between subjects and is relatively fast to calculate. It computes torsion from existing techniques such as tagging and HARP-tracking. Furthermore, it is investigated whether it is feasible to calculate rotation and torsion on a regional basis. By absence of a gold standard for torsion calculation, an analytical test case is used. The results obtained in healthy subjects are presented and compared to values from literature.

## Methods

### Calculation of rotation

The rotation of material points inside the LV myocardium at a certain level is calculated as the rotation *ϕ *around the AoR in a cardiac phase *t *relative to the previous cardiac phase using a cross product as follows:

(1) $$\phi (t)=\arcsin\left(\frac{A(t-1)\times A(t)}{\left|A(t-1)\right|\left|A(t)\right|}\right)$$

where

(2) $$A(t)=\left(\begin{array}{c} k_{\text{x}}(t)-c_{\text{x}}(t)\\ k_{\text{y}}(t)-c_{\text{y}}(t) \end{array}\right)$$

with *k *a point in the myocardium and *c *the AoR. |**A**(*t*)| is the radius of a point. After the rotation has been calculated for every point, the rotations are averaged to generate a global mean rotation *ϕ*_m_. The rotation *Φ *at a timeframe *n *is then set relative to the first timeframe as follows:

(3) $$\Phi (n)=\sum_{t=1}^{n}\phi_{\text{m}}(t)$$

### Calculation of torsion

The radius *ρ *of the ventricle at a certain level is calculated as the average of the radii of every myocardial point *k*. To calculate the torsion *T *at timeframe *t *as the mean CL shear angle from the rotation *Φ*, first the base rotation is subtracted from the apical rotation and divided by the distance *D *between the base and apex levels. This gives the twist per unit length. Then, the result is multiplied by the mean radius *ρ*_m _of the two levels and the first and the current cardiac phase, to take the mean radial location of the rotating point into account:

(4) $$\rho_{\text{m}}=\frac{\left(\rho_{apex}(t)+\rho_{apex}(1)+\rho_{base}(t)+\rho_{base}(1)\right)}{4}$$

(5) $$T(t)=\frac{\left(\Phi_{apex}(t)-\Phi_{base}(t)\right)\cdot \rho_{\text{m}}}{D}$$

### Regional rotation and torsion

**Figure 3:**
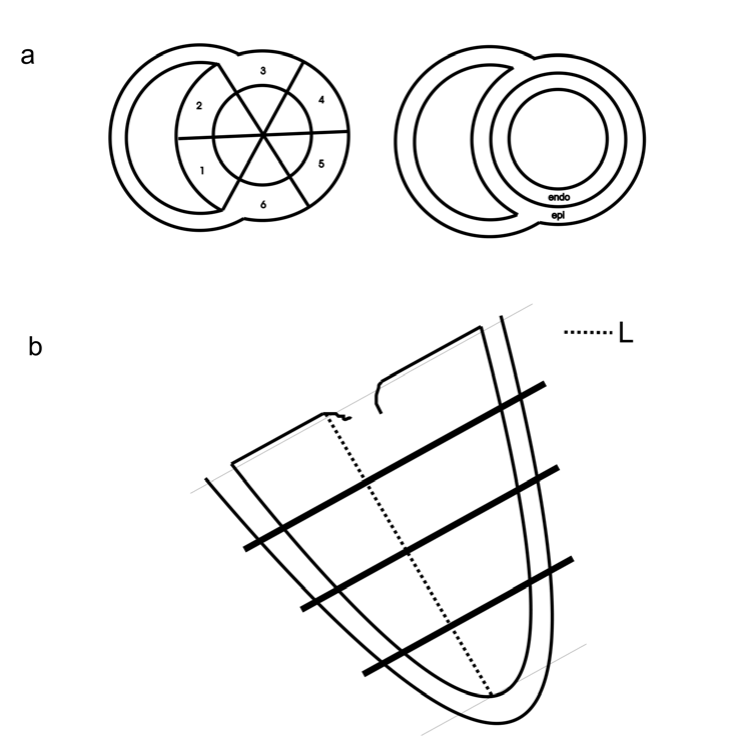
(a) Arrangement of the LV myocardial wall into circumferential segments (left), or transmural layers (right). (b) To localize the basal, mid and apical planes, the length (L) between the apex and the mitral valve in end systole is divided into four equal parts. The planes that are used for short-axis tagging are then located at a distance of 1/4, 1/2 and 3/4 of L from the mitral valve plane.

To calculate rotation or torsion on a regional basis, the myocardium was divided in circumferential segments and transmural layers (Fig. 3). For circumferential analysis, the rotation and the radius were averaged over the image points within circumferential segments. Torsion was calculated for each of the segments separately, using the same segments on the basal and apical level.

For transmural analysis, the endo- and/or epicardial contours that delineate the myocardium were shifted towards the desired transmural area. Then the rotation and the radius were averaged over the image points in the transmural layer and torsion was calculated as described above. Also in this case, the same transmural layers on the basal and apical levels were used.

### Analytical test case

To explore the influence of AoR location on the observed rotation and torsion, an analytical computer model test case was used [18]. Briefly, this was a 100 mm long incompressible deforming cylinder with a displacement given by analytical expressions. The deformed positions (*r*, *θ*, *z*) correspond to the undeformed positions (*R*, *Θ*, *Z*) as follows:

(6) $$r=\sqrt{r_{i}^{2}+(R^{2}-R_{i}^{2})}$$

(7) θ = ϕ*R *+ Θ + γ*Z *+ ε

(8) *z *= ω*R *+ *Z *+ δ

In this test case, the parameters were chosen such that human heart deformation was exaggerated (RC shear: ϕ = 0.556°/mm, CL shear: γ = 0.6°/mm, rigid body rotation: ε = 18.334°, RL shear: ω = 0.556, rigid body displacement: δ = 8.334 mm, initial outer radius: 50 mm, inner radii before and after deformation: R_i _= 25 mm, r_i _= 15 mm).

Rotation and torsion were calculated in circumferential segments and in a transmural layer using the test case. The test case had a fixed AoR over time, which was the center of the cylinder. The AoR was moved in all timeframes in order to test the sensitivity of the torsion value for small changes in AoR location. The AoR in the test case was displaced from the center of the cylinder between 0 and 20% of the mean radial length. This displacement, expressed as percentage, is indicated as the AoRdisp. The AoR was displaced separately for the slices on both ends of the cylinder in identical and opposite directions. Peak rotation and torsion were calculated in six circumferential segments and as a global mean (one transmural layer). In case of calculation in circumferential segments, the deviation per segment in rotation and torsion was averaged over all segments, and the results were presented as mean ± SD.

### Healthy subjects

In twelve healthy subjects (49 ± 11 yr, 3 female), rotation and torsion were calculated in circumferential segments and transmural layers, and as a global mean. The subjects had no history of cardiac disease, normal ECG, ejection fraction > 50% and blood pressures under 160/90 mmHg. Written informed consent was obtained from all volunteers. Circumferential analysis was performed in six segments: inferoseptal (IS), anteroseptal (AS), anterior (AN), anterolateral (AL), inferolateral (IL) and inferior (IN). Transmural analysis was performed in the endo- and epicardium (data from the endocardial 50% and the epicardial 50% of the myocardial wall were used).

### Image acquisition in the subjects

Tagged MR images were acquired on a 1.5T whole body MR scanner (Magnetom Sonata, Siemens, Erlangen, Germany). Sinusoidally, complementary spatial modulation of magnetization (CSPAMM) [19-21] short-axis cine MR images in two orthogonal directions were made with a SSFP sequence [22,23]. The temporal resolution was 14 ms, the FoV was 300 × 300 mm, the matrix size was 256 × 78, the slice thickness was 6 mm and the tag spacing was 7 mm (TR = 4.7 ms, TE = 2.3 ms, bandwidth = 369 Hz/pixel, flip angle = 20°). Prospective triggering and a multiple brief expiration breath hold scheme were used.

**Figure 4:**
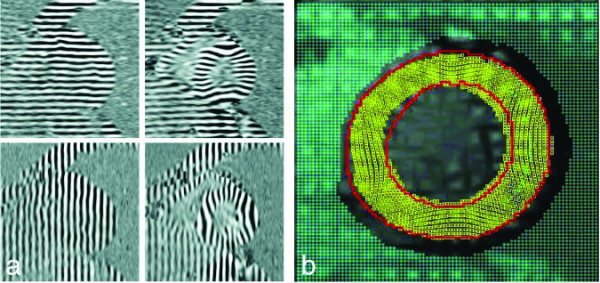
(a) Example of the horizontally and vertically tagged images for a healthy volunteer. Left: end-diastole, right: end-systole. Tags do not appear very sharp because they are sinusoidal, which is optimal for the HARP-tracking procedure. (b) Visualization of tracked points using the extended HARP tracking method (18). The red lines represent the myocardial contours, the yellow markers the tracked points.

Short-axis images were acquired at basal, mid and apical levels in the LV. The levels were determined by taking the distance between the apex-endocardium and the mitral valve plane in an end-systolic phase of a 2-chamber cine and dividing this distance into four equal parts (Fig. 3). With these sets of CSPAMM images (Fig. 4), automatic Harmonic Phase (HARP) tissue tracking was performed [18,24].

Additionally, a 3-chamber cine with the same high temporal resolution was acquired to determine the moment of aortic valve closure (AVC).

### LV contouring and extended HARP-tracking of subject data

In order to delineate the myocardium, harmonic-magnitude (HARM) images (magnitude image of the harmonic peak in the Fourier transform of a CSPAMM image) were calculated. LV endo- and epicardial contours were drawn semi-automatically on these images using a commercially available software package (Mass, Medis, Leiden, the Netherlands). Subsequently, automatic extended HARP tracking analysis [18] was applied using in house developed software. This method tracks all parts of the myocardium in all phases of the cardiac cycle with high spatial resolution, since it is not limited to tag line intersections (every pixel in the myocardium is considered to be a material point and is tracked, however, true spatial resolution is determined by the applied filtering and therefore lower than one pixel). Newly appearing material points that appear inside the contours as a result of longitudinal (through plane) motion are also tracked (Fig. 4).

### Rotation and torsion in subject data

With the displacement obtained from the tracking procedure, the rotation of the myocardium can be calculated for every tracked point inside the contours of the short-axis images. In this case it is assumed that the myocardium rotates around its center of mass. Therefore, the AoR is calculated from the mean of all image points inside the contours in every phase of the cardiac cycle. This means that the AoR is moving over time. The radius of the LV is calculated as the mean of all distances from every point in the myocardium to the AoR. As a result, the radius then also changes over time.

Torsion was analyzed between basal and apical slices (base-apex), and, in order to also provide longitudinal regional information, between basal and mid ventricular slices (base-mid), and mid and apical slices (mid-apex). Peak rotation, time to peak rotation, peak torsion and time to peak torsion were compared over circumferential segments (between segments with largest and smallest values and neighboring segments), transmural layers, and longitudinal levels using a paired Student's t-test. Correcting for multiple comparisons (Bonferroni), p-values below 0.003 were considered significant. The results are presented as mean ± SE.

## Results

### Analytical test case

**Figure 5:**
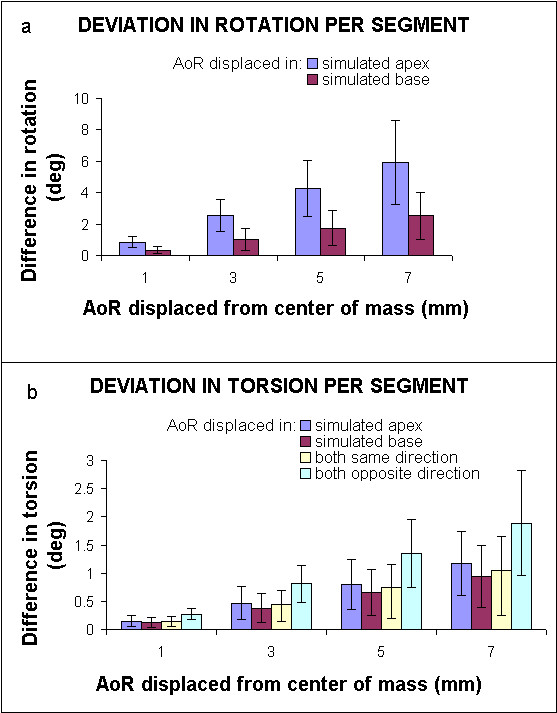
Circumferential rotation and torsion in the analytical test case: (a) Deviation in peak rotation per segment. (b) Deviation in peak torsion per segment. Z is the longitudinal axis of the cylinder. The mean global radius was 37.4 mm, but the radius per segment is used for the calculation of torsion. The simulated apex and base depict the largest and smallest Z in Eq. [5,6,7]. The maximum applied rotation at the simulated apex was 99.1°, and 39.7° at the simulated base. The error bars represent the SD of values among segments.

In Fig. 5, the results for circumferential rotation and torsion deviation for a displaced AoR are presented as the absolute average deviation over the regions ± SD. The observed deviation by moving the AoR in the slice on one end of the cylinder is larger than on the other end of the cylinder, since the overall rotation was larger in that slice per definition (Eq. [7]. The relative deviation in rotation or torsion from the analytical value as a function of AoRdisp can be found in Table 1.

**Table 1:** Analytical test case: Relative deviation of analytical peak rotation and torsion per AoRdisp*

	**Simulated apex displaced**	**Simulated base displaced**	**Both displaced in same direction**	**Both displaced in opposite direction**
**Torsion: global and transmural layers**	0.03	0.03	0.05	0.05
**Torsion: circumferential segments**	0.56 ± 0.27	0.45 ± 0.26	0.50 ± 0.29	0.90 ± 0.44
**Rotation: global and transmural layers**	0.001	0.002	n/a	n/a
**Rotation: circumferential segments**	0.64 ± 0.29	0.68 ± 0.40	n/a	n/a

*The simulated apex and base are the largest and smallest Z value (Eq. [5–7]) of the cylinder. Torsion and rotation calculated in circumferential segments are averaged over all segments.

For the displaced AoR in the test case, the relative error in the global torsion was below 0.05% per AoRdisp (Table 1). In contrast, the relative error in torsion in circumferential segments was up to 0.9% per AoRdisp.

### Healthy subjects

For the healthy subjects, peak and time to peak rotations (Table 2), and peak and time to peak torsion (Table 3) are given as mean ± SD. Peak values are given for global, transmural and circumferential calculations. Time to peak values are shown for global and transmural calculations only.

**Table 2:** Healthy subjects: Global and regional peak rotation (°) and global time to peak rotation (ms)*

	**Peak apex rotation**			**Peak mid rotation**		
**Segments**	**Total**	**Endo**	**Epi**	**Total**	**Endo**	**Epi**

**IS**	8.7 ± 3.2	9.2 ± 3.7	8.4 ± 3.1	3.5 ± 2.1	3.5 ± 2.1	3.5 ± 2.1
**AS**	12.4 ± 5.5	13.5 ± 5.7	11.3 ± 5.0	6.4 ± 1.8	6.8 ± 1.9	6.2 ± 1.8
**AN**	16.4 ± 7.7	17.5 ± 8.2	15.9 ± 7.5	6.9 ± 2.5	7.1 ± 2.6	6.6 ± 2.4
**AL**	15.7 ± 6.1	16.6 ± 6.3	15.5 ± 6.1	5.5 ± 2.4	5.8 ± 2.3	5.4 ± 2.4
**IL**	9.7 ± 3.9	9.8 ± 4.0	9.6 ± 3.6	4.8 ± 2.1	5.0 ± 2.2	4.7 ± 2.1
**IN**	7.3 ± 3.6	7.6 ± 4.1	7.4 ± 3.3	4.3 ± 1.8	4.4 ± 1.7	4.3 ± 1.8
**Global**	10.5 ± 3.4	11.0 ± 3.5	10.2 ± 3.3	3.8 ± 1.6	3.9 ± 1.6	3.8 ± 1.6
**Global timing**	384 ± 98	384 ± 98	387 ± 98	194 ± 94	194 ± 94	194 ± 94

	**Peak base rotation**			**Peak negative base rotation**		

**Segments**	**Total**	**Endo**	**Epi**	**Total**	**Endo**	**Epi**

**IS**	2.3 ± 2.0	2.3 ± 2.0	2.4 ± 2.0	-5.6 ± 4.1	-6.1 ± 4.3	-5.4 ± 4.0
**AS**	4.4 ± 2.1	4.6 ± 2.2	4.4 ± 2.1	-4.6 ± 2.3	-4.8 ± 2.4	-4.5 ± 2.2
**AN**	3.3 ± 1.8	3.3 ± 1.9	3.1 ± 1.7	-7.1 ± 3.5	-7.8 ± 4.0	-6.8 ± 3.0
**AL**	1.9 ± 1.3	1.9 ± 1.3	1.8 ± 1.3	-9.4 ± 3.7	-10.2 ± 4.0	-9.1 ± 3.7
**IL**	3.3 ± 1.6	3.4 ± 1.7	3.3 ± 1.5	-4.6 ± 2.2	-4.9 ± 2.3	-4.5 ± 2.2
**IN**	3.8 ± 3.0	3.8 ± 3.1	3.9 ± 3.0	-3.5 ± 3.0	-4.1 ± 3.4	-2.9 ± 2.7
**Global**	2.1 ± 1.2	2.1 ± 1.2	2.1 ± 1.2	-4.4 ± 1.8	-4.9 ± 1.8	-4.1 ± 1.8
**Global timing**	117 ± 19	108 ± 28	109 ± 28	377 ± 41	373 ± 42	378 ± 39

*"Global" is the whole circumference, "total" represents the endo- and epicardial regions together. It can be seen that rotation is largest in the anterolateral region, and smallest in the inferior region. This however does not hold for the positive peak in base and mid rotation, here anteroseptal is largest. Rotation is largest in the endocardial region. The endocardium reaches its peak rotation shortly before the epicardium. IS: inferoseptal, AS: anteroseptal, AN: anterior, AL: anterolateral, IL: inferolateral, IN: inferior.

**Table 3:** Healthy subjects: Global and regional peak torsion (CL shear angle (°)) and global time to peak torsion (ms)*

	**BASE-APEX**			**BASE-MID**			**MID-APEX**		
**Segments**	**Total**	**Endo**	**Epi**	**Total**	**Endo**	**Epi**	**Total**	**Endo**	**Epi**

**IS**	6.7 ± 3.3	6.6 ± 3.2	6.9 ± 3.6	8.6 ± 4.3	8.5 ± 4.1	9.0 ± 4.5	8.8 ± 4.5	8.6 ± 4.3	9.3 ± 4.9
**AS**	8.3 ± 3.2	8.2 ± 3.1	8.3 ± 3.3	10.4 ± 3.6	10.0 ± 3.6	10.7 ± 4.1	10.1 ± 5.8	10.0 ± 5.5	10.0 ± 5.8
**AN**	11.7 ± 4.7	11.4 ± 4.6	12.2 ± 4.7	13.3 ± 6.1	13.3 ± 6.0	13.3 ± 6.0	13.5 ± 5.5	13.1 ± 5.5	14.4 ± 5.8
**AL**	12.4 ± 3.9	11.9 ± 3.7	13.1 ± 4.0	14.8 ± 4.4	14.6 ± 4.3	15.4 ± 4.6	13.5 ± 5.6	12.8 ± 5.3	14.5 ± 6.1
**IL**	6.5 ± 2.5	5.9 ± 2.5	6.8 ± 2.5	9.3 ± 4.2	9.2 ± 4.2	9.8 ± 4.4	8.0 ± 4.5	7.1 ± 4.4	8.4 ± 4.5
**IN**	5.0 ± 3.3	5.0 ± 3.4	5.0 ± 3.2	7.7 ± 4.2	7.9 ± 4.1	7.9 ± 4.4	6.7 ± 3.6	6.5 ± 3.5	7.0 ± 3.8
**Global**	7.7 ± 1.4	7.5 ± 1.3	8.0 ± 1.5	8.2 ± 2.3	8.2 ± 2.2	8.3 ± 2.3	8.1 ± 1.1	7.6 ± 1.1	8.5 ± 1.3
**Global timing**	366 ± 24	366 ± 32	367 ± 25	357 ± 33	356 ± 32	355 ± 33	370 ± 28	369 ± 26	367 ± 25

*"Global" is the whole circumference, "total" represents the endo- and epicardial regions together. It can be seen that torsion is largest in the anterolateral region, and smallest in the inferior region. Torsion is also largest in the epicardial region. Peak base-mid torsion occurs before peak mid-apex torsion. IS: inferoseptal, AS: anteroseptal, AN: anterior, AL: anterolateral, IL: inferolateral, IN: inferior.

### Circumferential segments in subjects

**Figure 6:**
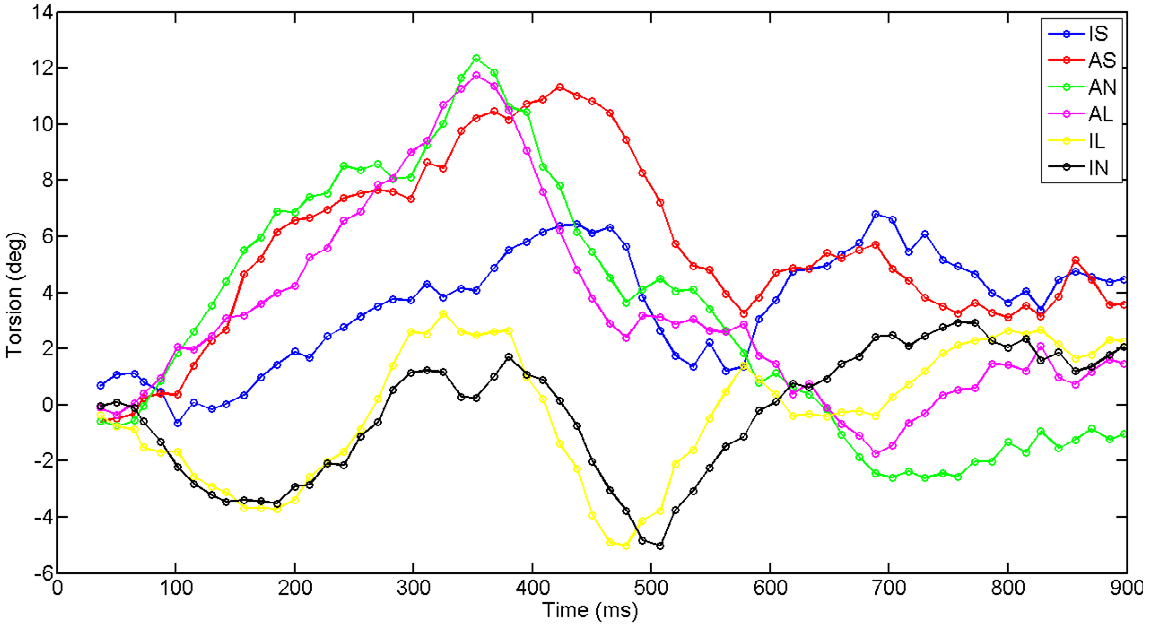
Segmental base-apex torsion in a healthy volunteer. Counterclockwise torsion is positive when viewed from the apex. IS: inferoseptal, AS: anteroseptal, AN: anterior, AL: anterolateral, IL: inferolateral, IN: inferior.

When rotation and torsion are calculated in circumferential segments, differences in peak values and timing of peak values are present (Fig. 6, Tables 2, 3). Anterior/anterolateral rotation and torsion were larger than inferior/inferoseptal. However, no significant differences between segments were found in peak values (p = 0.01) or time to peak values (p > 0.1).

### Transmural layers in subjects

**Figure 7:**
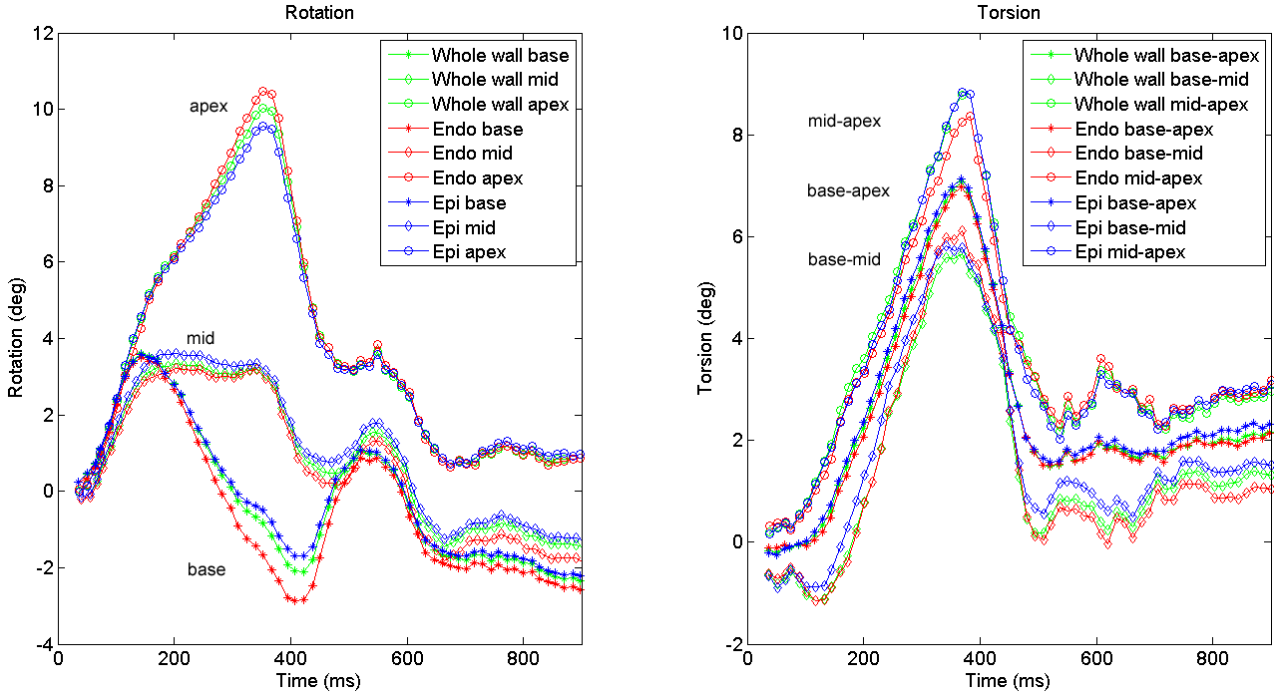
Rotation and torsion in a healthy volunteer. The different colors divide transmural layers; the different shapes indicate different longitudinal levels. Transmural and longitudinal differences are visible in this subject. Positive rotation is counterclockwise when viewed from the apex. Note that in this subject, torsion is not constant over the long axis.

**Figure 8:**
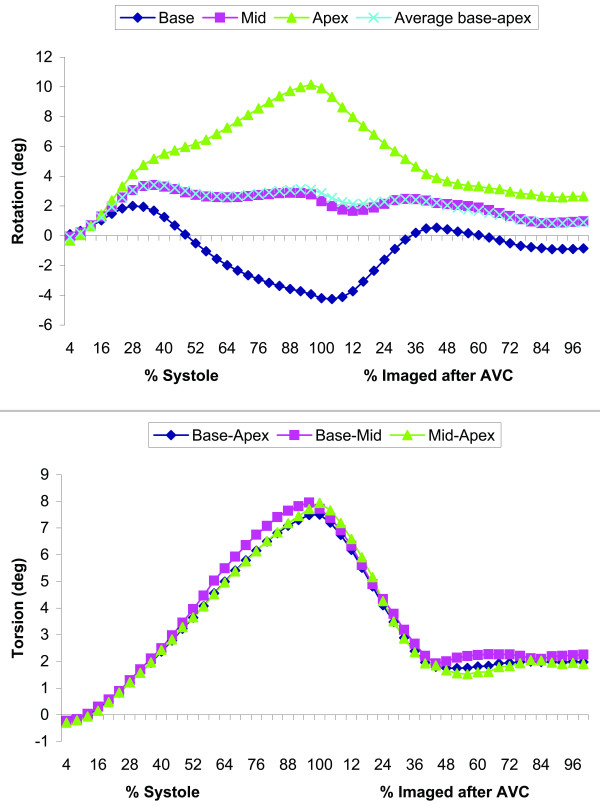
Mean, interpolated rotation (a) and torsion (b) over all 12 healthy subjects. 100% systole is defined as AVC. The remaining parts of the curves (imaged after AVC) are also interpolated over a 100% range, note that this is not the entire diastolic period. The calculated average of base and apex rotation is nearly the same as the measured mid rotation.

In Fig. 7, transmural basal, mid and apical rotations and transmural base-apex, base-mid and mid-apex torsion are shown for the same subject. It can be seen that the endocardium rotates more than the epicardium. The base starts rotating similar to the apical slice, but subsequently rotates in the opposite direction. Besides the first, positive peak, this results in a negative peak value at the basal level. The rotation in the mid-slice follows an averaged path between base and apex. The mean rotation over all subjects is shown in Fig. 8. The torsion demonstrates a comparable pattern for all combinations between the slices (Fig. 8).

Transmural differences (Table 2, 3) in peak rotation and peak torsion were significant for all calculated values on the basal and apical level (all p < 0.001), except for peak positive base rotation. No significant difference was found between base-mid and mid-apex peak torsion (p = 0.9).

Transmural (p > 0.05) and longitudinal (p = 0.03) differences in timing were not significant. Also, no significant difference was found between AVC and time to peak torsion (p > 0.2).

## Discussion

This study demonstrates that the calculation of LV rotation and torsion in circumferential segments using an AoR provides unreliable results, since these results are strongly dependent on the location of the AoR. Through the availability of displacement data with high spatial resolution, LV rotation and torsion could also be calculated transmurally. These results are hardly dependent on AoR location.

### Displaced AoR in the test case

The influence of a displaced AoR on the observed rotation and torsion in the analytical test case appears to be larger when the AoR was moved in a slice where the actual rotation was larger (Fig. 5). However, when looking at the percent deviation (Table 1), the percentage from the analytical value is higher in a slice with a small rotation than in a slice with a large rotation. This suggests, that in a human heart (where the rotation is smaller than in the exaggerated test case), the relative deviation in rotation from a displaced AoR will increase even more at increasing AoRdisp. For torsion, displacement of the AoR in the slice with the largest original rotation results in the largest error at increasing AoRdisp (Table 1). This is caused by the fact that torsion has one analytical value for both combined slices (unlike rotation, where both separate slices have different analytical rotations). This value is used for the error calculation for AoR displacements in both slices. In case of calculation in circumferential segments, the deviation in observed rotation is hundreds of times larger than in the global calculation.

For torsion calculation, the radius of each segment is taken into account, which reduces the rotational effect (Fig. 2) of the displaced AoR a little. However, observed deviations in segmental torsion from a displaced AoR are still up to 25 times higher than for a global calculation of torsion. From the analytical test case, it can be concluded that calculating rotation and torsion in circumferential segments is very much influenced by the position of the AoR and is therefore unreliable.

In the transmural calculation of rotation and torsion, the influence of AoR location was not found to be of critical importance. For example, in case of a 10% AoRdisp (which generally corresponds to a displacement of about 2.5 mm in a normal human heart) caused by inadequate contouring, the deviation calculated in circumferential segments would be up to 0.9 ± 0.4° on an average torsion of 10°. For a calculation in a global or transmural segment, this deviation would be no more than 0.05°. AoRdisp might be even larger in case of local heart wall thickening, for example.

### Healthy subjects

In the subjects, the transition in rotation direction between base and apex proceeds gradually (Figs. 7, 8). This was in line with previous reporting [16]. Mid-slice rotation is almost the exact average of base and apex rotation over the entire measurement period (Fig. 8). This implies that the change in LV rotation is very evenly distributed over the long axis of the LV. The gradient in LV rotation causes the constant torsion over the whole LV (Fig. 8). However, torsion was not constant over the LV in every individual subject (e.g. Fig. 7). The mechanism that causes the whole LV to rotate clockwise initially remains to be investigated. Ingels et al. [25] suggested an explanation for the direction of torsion during the systolic period, by describing how the transmurally different obliquely oriented myofibers are subsequently activated and cause torsion, but does not speculate about the cause of the rotational directions.

### Torsion in circumferential segments in the subjects

In several studies it was found that the septal and inferior wall show less torsion than the anterior and lateral wall [11-13,15,16]. The difference between these segments appears to be around 50% in literature, but was not always found to be statistically significant. This study is in line with this finding.

In previous studies, torsion was measured under different definitions of the AoR. The AoR was either chosen as a moving center of the LV [15,16], or was fixed over time [11,12]. Nevertheless, the AoR was always placed somewhere near the center of the LV, resulting in a difference of around 50% between the IS and AL locations. Moreover, the definition of torsion (Fig. 1) is not always the same, and not all studies used human subjects, which makes a comparison between studies difficult.

### Definition of the AoR

Our findings are very much consistent with the study of Lorenz et al. [16], who also used a moving LV center of mass as AoR. Only our circumferential peak torsion (CL shear angle) values are somewhat larger, which may be the result of their measuring at 80% of systole.

The variance between segments could have a physiological origin, or could be caused by the fact that the septum is more fixed to the RV and therefore can rotate less. However, if this were the cause, one would expect also a lower anteroseptal/anterior value. Segmental differences caused by errors in AoR location might be more random than segmental differences of physiological origin. The large variance in segmental values found in this study therefore suggests that the observed differences are not physiological, but caused by a deviation in the AoR. Small variations between segments (up to 10%) might be caused by only a slight error in AoR position (e.g. miscontouring). For larger variations, it seems likely that the choice of the AoR is wrong, and that the RV mass plays a role in the process of torsion, such that the heart rotates around its total center of mass. It can be seen in a short axis image that the majority of RV mass is located in its inferolateral region, which would move the combined center of mass more towards inferoseptal, as seen from the LV (Fig. 2). This would perhaps balance circumferential regional rotation more.

### Clinical implications

Since relatively large variations in circumferential regional torsion at only small variations in AoR position were found in the test case, and regarding the above mentioned limitations of the segmental analysis of rotation and torsion in human volunteers, it seems secure to calculate rotation and torsion over the whole circumference only. Therefore, torsion values from circumferential segments should be interpreted with great awareness. Moreover, considering fiber orientation and its relation to torsion, torsion seems to be a more global measure. An abnormality originating at a certain circumferential location will influence torsion in the whole heart due to the connection of the myofibers. For example, local ischemia was found to influence torsion on multiple locations [17]. Therefore, it seems more interesting to focus on the obliquely oriented endo- and epicardial fibers, which cause the heart to twist, and study transmural variations in torsion. Since abnormalities in myocardial function are found to express themselves via the torsion [3-9,26-28], it might be a suitable tool for early detection of for instance subclinical heart failure.

The fibers in the cardiac wall are known to vary in orientation in the transmural direction. These fiber orientations are well described by the ventricular-myocardial band concept [29] which was confirmed by several studies using diffusion tensor imaging fiber tracking CMR [30,31]. In this study, the cardiac wall was divided into two equal segments, an endo- and an epicardial segment, which should cover the most important fiber directions (ascending and descending) causing the torsional deformation.

### Torsion in transmural layers in the subjects

**Figure 9:**
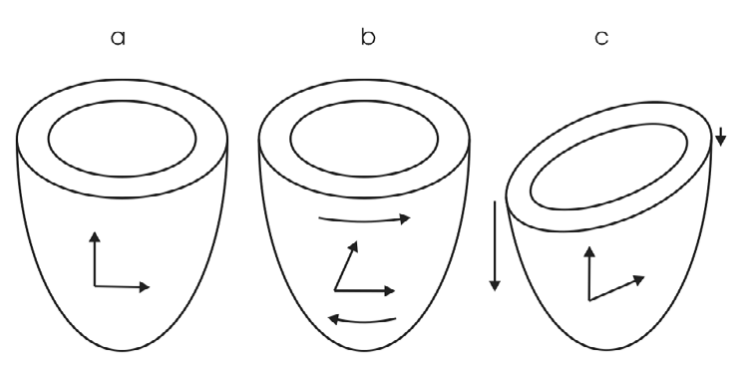
Two modes of deformation resulting in the same CL shear angle [33]. a) Undeformed LV, b) LV deformed due to torsion, c) LV deformed due to differences in longitudinal displacement.

In line with the studies of Buchalter et al. [11] and Young et al. [15], we found that in the endocardium, rotation was larger than in the epicardium. However, our finding that endocardial torsion was smaller than epicardial torsion is in contrast to the study of Buchalter et al., who found a constant CL shear angle through the wall. In a canine study of Prinzen et al. [32], endocardial torsion (CL shear) was found to be higher than epicardial torsion in a control group. These results may be due to a different definition of the radius for calculating the CL shear angle (Buchalter et al. did not include the basal slice for determining the radius and the rotation), or to the use of a 3D strain tensor for the calculation, which is a different calculation method. However, in this 3D approach, information about torsion in circumferential segments will also not be reliable. That is, differences in the regional circumferential-longitudinal shear angle can also result from longitudinal displacement (Fig. 9). This is an effect that cancels out when the local CL shear is averaged over the whole circumference. To compare the proposed torsion calculation method with the local CL shear angle as computed by a 3D strain tensor, which is very time consuming, is part of our current research.

## Conclusion

It was shown that the analysis of torsion in circumferential segments is unreliable. Analysis in transmural layers however, was found to be accurate and of great interest for further exploration of the mechanics of contraction and relaxation in the left ventricle. Torsion could be easily calculated by the proposed, comparable method in healthy subjects and was in conformity with previous studies. The transmural results from the healthy subjects can be used as reference data for future patient studies. With the obtained knowledge, torsion data from circumferential segments should be interpreted as unreliable. The proposed method to calculate torsion may be used to provide more understanding in cardiac function and it may serve as a tool for early detection of subclinical heart disease.

## Competing interests

The authors declare that they have no competing interests.

## Authors' contributions

IKR Study design, data analysis and interpretation, manuscript preparation, MJG Study design, manuscript revision, JPK Data interpretation, manuscript revision, JTM Data collection, manuscript revision. All authors read and approved the manuscript.
